# Comparison of single-trait and multiple-trait genomic prediction models

**DOI:** 10.1186/1471-2156-15-30

**Published:** 2014-03-04

**Authors:** Gang Guo, Fuping Zhao, Yachun Wang, Yuan Zhang, Lixin Du, Guosheng Su

**Affiliations:** 1 National Center for Molecular Genetics and Breeding of Animal, Institute of Animal Sciences, Chinese academy of Agricultural Sciences, Beijing 100193, China; 2 Beijing Sanyuan Lvhe Dairy Cattle Breeding Center, Beijing 100076, China; 3 College of Animal Science and Technology, China Agricultural University, Beijing 100193, China; 4 Department of Genetics and Biotechnology, Faculty of Agricultural Sciences, Aarhus University, Tjele DK-8830, Denmark

**Keywords:** Genomic selection, Reliability, Multiple-trait model, Single-trait model, Heritability

## Abstract

**Background:**

In this study, a single-trait genomic model (STGM) is compared with a multiple-trait genomic model (MTGM) for genomic prediction using conventional estimated breeding values (EBVs) calculated using a conventional single-trait and multiple-trait linear mixed models as the response variables. Three scenarios with and without missing data were simulated; no missing data, 90% missing data in a trait with high heritability, and 90% missing data in a trait with low heritability. The simulated genome had a length of 500 cM with 5000 equally spaced single nucleotide polymorphism markers and 300 randomly distributed quantitative trait loci (QTL). The true breeding values of each trait were determined using 200 of the QTLs, and the remaining 100 QTLs were assumed to affect both the high (trait I with heritability of 0.3) and the low (trait II with heritability of 0.05) heritability traits. The genetic correlation between traits I and II was 0.5, and the residual correlation was zero.

**Results:**

The results showed that when there were no missing records, MTGM and STGM gave the same reliability for the genomic predictions for trait I while, for trait II, MTGM performed better that STGM. When there were missing records for one of the two traits, MTGM performed much better than STGM. In general, the difference in reliability of genomic EBVs predicted using the EBV response variables estimated from either the multiple-trait or single-trait models was relatively small for the trait without missing data. However, for the trait with missing data, the EBV response variable obtained from the multiple-trait model gave a more reliable genomic prediction than the EBV response variable from the single-trait model.

**Conclusions:**

These results indicate that MTGM performed better than STGM for the trait with low heritability and for the trait with a limited number of records. Even when the EBV response variable was obtained using the multiple-trait model, the genomic prediction using MTGM was more reliable than the prediction using the STGM.

## Background

The availability of genome-wide markers, such as single nucleotide polymorphism (SNP) markers, has made it possible to predict breeding values of candidate animals using genomic information. The genomic prediction principle was first proposed by Meuwissen *et al.*[1]. A typical genomic prediction procedure is to estimate simultaneously the effects of all the SNPs available in the genotype data, and then to sum up all the predicted SNP effects as the genomic estimated breeding value (GEBV). Selection based on the GEBV is called genomic selection. Because GEBV is calculated based on genetic marker information rather than on phenotypic information, genomic selection can shorten the generation interval, while maintaining the accuracy of the estimated breeding value (EBV) at an acceptable level [1,2]. Genomic selection is especially useful for low heritability traits, sex-limited traits, and traits that are difficult or expensive to measure, such as carcass, health, longevity, and fertility traits. The advantages of genomic selection have been corroborated by simulation and empirical studies [1-5]. Recently, genomic selection has been successfully implemented in dairy cattle breeding programs in many countries to accelerate the genetic progress and reduce the cost of progeny testing [6-9].

Numerous genomic selection studies have focused on single-trait analyses. However, many traits are genetically correlated, such as the reproductive and milk yield traits in dairy cattle and these traits may have different heritabilities. Some traits, such as feed efficiency, may be recorded only in a small number of animals because of the difficulty of measuring them. Like traditional genetic evaluation, a multiple-trait model is expected to increase the accuracy of the GEBV by making use of information from genetically correlated traits. The benefit of using a multiple-trait model will be more profound for traits with low heritability and a small number of phenotypic records. Multiple-trait models for genomic prediction have been reported recently [10-14]. It has been shown that a multiple-trait genomic model (MTGM) had higher prediction accuracy than a single-trait genomic model (STGM).

Bayesian variable selection methods generally outperform linear mixed models; often called the genomic best linear unbiased prediction (GBLUP) method [1,15]. However, the advantage of Bayesian methods over GBLUP is dependent on the genetic architecture [16], such as the number of quantitative trait loci (QTLs) and the density of the markers. Clark *et al.*[17] found that GBLUP produced slightly better prediction accuracy than the BayesB model when a trait was affected by a large number of QTLs with small effects. Similar trends were observed by Coster *et al.*[18] and Li *et al.*[19]. Many studies using real data have reported that GBLUP performs as well as Bayesian variable selection models for most traits [20-22]. Ober *et al.*[23] showed that BayesB was less accurate than GBLUP in predicting phenotypes of QTLs based on the genomic sequence data of *Drosophila melanogaster*. The main advantages of GBLUP over Bayesian methods are, its implementation is straightforward using existing residual maximum likelihood (REML) and BLUP programs, and it requires less computation time, which can be an important factor in the practical application of a genomic prediction method. Although some studies have shown that computing time for Bayesian models can be reduced greatly using the expectation–maximization (EM) and variational Bayes algorithms [19,24], GBLUP models (at either the SNP or individual animal levels) are still attractive approaches in practical genomic evaluations [6,7,25].

Three types of response variables that have been used widely to predict the GEBV are EBV, daughter yield deviation (DYD), and deregressed proof (DRP) [9,13,14,26]. The EBV of a bull is calculated from the information of all available relatives including the daughters. The DYD of a bull is the average of the daughters’ actual performances adjusted for fixed and non-genetic random effects and genetic effects of the daughters’ dams. The DRP is derived from the EBV [27] and can be considered as an analogue of DYD. Because EBV is estimated from the information of all relatives, the reliability of EBV is higher than that of DYD or DRP. Furthermore, EBV can be obtained directly from a database of routine genetic evaluations. Our previous simulation study showed that, in genomic prediction, using the conventional EBV as the response variable gave slightly better results than using DYD in most scenarios [26]. In practical routine genetic evaluation, EBV (and also DRP or DYD) is usually calculated using multiple-trait models. This poses an important question: are MTGMs needed if a multiple-trait model is used to derive the response variables?

The objective of this study was to compare a STGM and a MTGM for genomic prediction using conventional EBVs estimated with a conventional single-trait linear mixed model and a conventional multiple-trait linear mixed model as response variables. The comparison was carried out using data from various simulation scenarios considering heritability of two genetically correlated traits and the proportion of missing records in the data for the two traits.

## Materials and methods

### Simulation schemes

Genomic predictions were obtained using both a STGM and MTGM with simulated data for two genetically correlated traits. Trait I was assumed to have high heritability (h^2^ = 0.3) and trait II was assumed to have low heritability (h^2^ = 0.05). The genetic correlation was set as 0.5 and the residual error correlation was 0.

In the simulation scheme, the initial population comprised 50 sires and 50 dams, and this structure was kept constant for 50 historical generations. Then the population was extended to 1,000 sires and 200,000 dams. Thereafter, four generations (G1–G4) were generated to obtain the data used for the analysis. The population was assumed to be under random mating conditions with no overlapping generations. In G1–G4, all the bulls were genotyped and all the cows had a phenotypic record. The G1–G3 bulls were used as “reference animals” and their EBVs were used as the response variables for the genomic predictions; the G4 bulls were used as “validation animals”.

The simulations also generated reference populations with a small amount of data for one of the two traits. To simplify the simulation and analysis without losing the generality of the data, traits with a small number of records were handled by masking the EBVs of some individuals in the reference population, instead of by generating incomplete phenotype data. In this way, three response variable datasets were generated: (1) no missing EBVs for either of the traits; (2) no missing EBVs for trait II, but 90% of the EBVs for trait I were missed at random (*i.e.* the EBVs of only 300 of the 3000 bulls in the reference population were used for genomic prediction); and (3) no missing EBVs of trait I, but 90% of the EBVs for trait II were missed at random.

The simulated genome consisted of five chromosomes (100 cM each). A total of 5000 biallelic SNP markers were spaced equally across the whole genome at distances of 0.1 cM. Three hundred biallelic QTLs were generated and divided randomly into three groups (100 each). One hundred of the QTLs affected only trait I, 100 affected only trait II, and 100 affected traits I and II. The QTL positions were located randomly according to the gene distribution in the first 500 cM of the standard mouse genome (NCBI 2005). The QTL effects were drawn from a gamma distribution with a shape parameter of 0.84 and scale parameter of 5.4, and assigned positive or negative by equal chance. Hayes and Goddard [28] noted that published estimates of QTL effects resembled a gamma distribution with shape parameter of 0.4. However, generally, only the QTLs that are statistically significant are reported in the literature. This conditional reporting can lead to a marked upward bias of the shape parameter [29]. Therefore, in the present study, a more representative shape parameter of 0.64 was chosen. A scale parameter of 5.4 was chosen arbitrarily because the variance of the resulting EBVs was standardized before use. The 100 pleiotropic QTLs were assumed to have the same effect for both traits; therefore, the expected genetic correlation between the two traits is 0.5. All QTL effects were assumed to be additive. True breeding values (TBV) were calculated by summing all the QTL effects and subsequently scaling them to a realized genetic variance of 1. Phenotypic value was generated as the sum of the TBVs and a random residual sampled from a normal distribution *N*(0, (1-*h*^*2*^)/*h*^*2*^).

### Statistical models

The GEBVs were predicted using the performance and genotype information of the bulls in the reference population. The genomic prediction model used in this study was GBLUP, which is a linear mixed model with a genomic relationship matrix.

The STGM is defined as:

(1) y=1μ+Zg+e

where **y** is the vector of response variables (the conventional EBV was used in this study), **1** is the vector with elements of 1, μ is the intercept, **g** is the vector of genomic breeding values, **Z** is the design matrix that associates genomic breeding values with response variables, and **e** is the vector of random residuals. It is assumed that $g\sim N\left(0,G\sigma_{g}^{2}\right)$, where $\sigma_{g}^{2}$ is additive genetic variance and **G** is the realized relationship matrix calculated from the SNP marker information, and $e\sim N\left(0,I\sigma_{e}^{2}\right)$, where $\sigma_{e}^{2}$ is residual variance and **I** is the *n* × *n* identity matrix. A detailed description of how **G** is computed can be found in VanRaden [30] and Hayes *et al.*[6].

The MTGM is defined as:

(2) $$\left[\begin{array}{c} y_{1}\\ y_{2} \end{array}\right]=\left[\begin{array}{cc} I_{1} & 0\\ 0 & I_{2} \end{array}\right]\;\left[\begin{array}{c} \mu_{1}\\ \mu_{2} \end{array}\right]+\left[\begin{array}{cc} Z_{1} & 0\\ 0 & Z_{2} \end{array}\right]\;\left[\begin{array}{c} g_{1}\\ g_{2} \end{array}\right]+\left[\begin{array}{c} e_{1}\\ e_{2} \end{array}\right]$$

where $\left[\begin{array}{c} y_{1}\\ y_{2} \end{array}\right]$ is the vector of response variables of traits I and II, *I*_1_ and *I*_2_ are the identity matrices, $\left[\begin{array}{c} \mu_{1}\\ \mu_{2} \end{array}\right]$ is the vector of intercepts of traits I and II, $\left[\begin{array}{c} g_{1}\\ g_{2} \end{array}\right]$ is the vector of genomic breeding values of the two traits, **Z**_**1**_ and **Z**_**2**_ are the design matrix that associate genomic breeding values with response variables, and $\left[\begin{array}{c} e_{1}\\ e_{2} \end{array}\right]$ is the vector of random residuals of the two traits. It is assumed that $\left[\begin{array}{c} g_{1}\\ g_{2} \end{array}\right]\sim N\left(0,G⊗H\right)$, where $H=\left[\begin{array}{cc} \sigma_{g_{1}}^{2} & \sigma_{g_{12}}\\ \sigma_{g_{12}} & \sigma_{g_{2}}^{2} \end{array}\right]$ is the variance and covariance matrix of the genomic breeding values of the two traits, and $\left[\begin{array}{c} e_{1}\\ e_{2} \end{array}\right]\sim N\left(0,I⊗R\right)$, where $R=\left[\begin{array}{cc} \sigma_{e_{1}}^{2} & \sigma_{e_{12}}\\ \sigma_{e_{12}} & \sigma_{e_{2}}^{2} \end{array}\right]$ is the residual variance and covariance matrix of the two traits.

Although DRP is usually used as the response variable for genomic predictions, in this study, conventional EBVs were used to omit the extra calculation of DRP but without losing the generality of the comparisons between the different scenarios. The EBVs of the animals were estimated from phenotypic data of all the dams in G1–G4 using both a single-trait and a multiple-trait animal model. The models for estimating EBVs were consistent with the genomic prediction models described above, but incorporating a pedigree-based genetic relationship matrix, instead of a genomic relationship matrix. The variance components were estimated from the data and used to calculate the EBVs and GEBVs using the average information REML algorithm (AI-REML). The analysis was executed using the DMU package [31].

### Evaluation of genomic prediction

The evaluation was based on 10 replicates for each scenario and the average of the results was reported. The positions and effects of the QTLs were randomized, and the initial population was generated separately for each replicate. For each scenario, the reliability of the genomic predictions was measured as the squared correlation between the GEBVs and the TBVs ($R_{\mathrm{GEBV}}^{2}$). Unbiasedness was assessed by regression of the TBVs on the GEBVs. Because EBV is a regressed variable, using EBV as the response variable for genomic prediction will deflate the GEBV in proportion to the reliability of the EBV. GEBV in the real scale can be obtained by scaling GEBV with 1/$r_{\mathrm{EBV}}^{2}$, where $r_{\mathrm{EBV}}^{2}$ is the average reliability of EBVs of the animals in the reference population[26]. Therefore, the regression coefficients were calculated based on the original GEBV and the rescaled GEBV. A Hotelling-Williams *t* test [32,33] was used to determine the difference between the validation correlations obtained from the single-trait and multi-trait models.

## Results

### Reliability of EBV and regression coefficient of TBV on EBV

For the animals with no records, the EBVs were predicted from the information of their relatives. For the validation animals, the EBV for trait I (h^2^ = 0.30) had much higher reliability than the EBV for trait II (h^2^ = 0.05), as shown in Table 1. The multiple-trait model did not improve the reliability of the EBV for trait I, but significantly increased the prediction accuracy of the EVB for trait II. In addition, the multiple-trait model slightly improved unbiasedness for trait II because, for trait II, the regression coefficient was close to 1; however, the regression coefficients between the single-trait and multiple-trait models were not statistically different.

**Table 1:** Reliability of estimated breeding values (EBVs) and regression coefficients of true breeding value on EBV (The subscripts are the standard deviations of 10 replicates)

**Trait**	**Reference animals**		**Validation animals**			
	**Single-trait**	**Multiple-trait**	**Single-trait**		**Multiple-trait**	
	$R_{\mathrm{EBV}\_s}^{2}$	$R_{\mathrm{EBV}\_m}^{2}$	$R_{\mathrm{EBV}\_s}^{2}$	*b*_*EBV* _ *s*_	$R_{\mathrm{EBV}\_m}^{2}$	*b*_*EBV* _ *m*_
I	0.951_0.001_	0.952_0.001_	0.348_0.023_	0.970_0.034_	0.350_0.024_	0.971_0.037_
II	0.792_0.004_	0.822_0.014_	0.182_0.009_	0.905_0.074_	0.229_0.019_	0.919_0.071_

$$R_{\mathrm{EBV}}^{2}$$

: the squared correlation between conventional EBVs and true breeding value (TBV) for the reference and validation animals;

*b*_*EBV*_: the regression coefficient of TBV on the EBV for the validation animals;

*EBV*_*s*: EBV response variable estimated using the conventional single-trait linear mixed model;

*EBV*_*m*: EBV response variable estimated using the conventional multi-trait linear mixed model.

### Reliability of GEBV

The reliabilities of the GEBVs for animals in the validation population are presented in Table 2. When no trait records were missing, MTGM and STGM generated the same reliability for trait I, while for trait II, MTGM increased the reliability of the GEBV by 0.007 when using the EBV response variable generated by the multi-trait model (EBV_m) and by 0.033 when using the EBV response variable generated by the single-trait model (EBV_s). When there were missing records for trait I, the reliability of GEBV was 0.142 higher with MTGM than with STGM using EBV_m and 0.111 higher using EBV_s. When there were missing records in trait II, the reliability of GEBV was 0.181 higher with MTGM than with STGM using EBV_m and 0.211 higher using EBV_s. Moreover, the reliability of the GEBVs generated with the same model using EBV_m as the response variable was higher than their reliability using EBV_s in all scenarios with missing records.

**Table 2:** Reliability of genomic estimated breeding values (GEBVs) for the validation animals (The subscripts are the standard deviations 10 replicates)

**Trait**	**Data type**	**Single-trait**		**Multiple-trait**	
		$R_{\mathrm{GEBV}\_m}^{2}$	$R_{\mathrm{GEBV}\_s}^{2}$	$R_{\mathrm{GEBV}\_m}^{2}$	$R_{\mathrm{GEBV}\_s}^{2}$
I	No missing for both traits	0.874_0.003_	0.873_0.002_	0.874_0.001_	0.873_0.002_
	Missing for trait I	0.474_0.006_	0.473_0.008_	0.616_0.004_	0.584_0.004_
II	No missing for both traits	0.718_0.007_	0.723_0.006_	0.725_0.005_	0.756_0.006_
	Missing for trait II	0.373_0.010_	0.338_0.013_	0.554_0.009_	0.549_0.011_

$$R_{\mathrm{GEBV}}^{2}$$

: the squared correlation between GEBV and true breeding value (TBV) for the validation animals using the single-trait and multi-trait models;

*G*_*EBV*_*m*_: GEBV predicted using the EBV response variable estimated using the conventional multi- trait linear mixed model;

*G*_*EBV*_*s*_: GEBV predicted using the EBV response variable estimated using the conventional single trait linear mixed model.

The Hotelling Williams *t* test showed that the accuracy of genomic prediction using EBV_m was significantly higher than the accuracy using EBV_s for traits with missing records (Table 3). The differences in accuracy between the single-trait and multi-trait models were not statistically significant in the scenarios with no missing records.

**Table 3:** **t values of Hotelling Williams *t *** **test in difference between correlations (correlation between genomic prediction and true breeding value) from the single- trait and the multiple-trait models**

**Trait**	**Data type**	**Hotelling Williams t value**	
		**GEBV**_**m**	**GEBV**_**s**
I	No missing for both traits	0.000	0.000
	Missing for trait I	7.156**	6.237**
II	No missing for both traits	0.718	2.701
	Missing for trait II	8.079**	8.883**

**Significantly different (P > 0.01).

GEBV_m: the genomic estimated breeding value (GEBV) predicted using the EBV response variable estimated from the conventional multi-trait linear mixed model.

GEBV_s: the GEBV predicted using the EBV response variable estimated from the conventional single-trait linear mixed model.

### Regression of TBV on GEBV

Table 4 shows the regression coefficients of TBV on GEBV in the validation data. For all scenarios, the intercept was close to 0 (in the range -0.018 to 0.012, data not shown). Before rescaling the GEBVs the regression coefficients (b_GEBV_m_ and b_GEBV_s_) were greater than 1 for all scenarios, and this was more serious for trait II (low heritability) than for trait I (high heritability). After rescaling the GEBVs (by dividing the GEBVs by the average reliability of the EBVs), the newly calculated regression coefficients (b_GEBV_mc_ and b_GEBV_sc_) ranged from 0.832 to 1.002 (with an average of 0.907) using EBV_m, and from 0.964 to 1.069 (with an average of 1.021) using EBV_s. In general, the differences between the regression coefficients for STGM and MTGM using the same response variables were small.

**Table 4:** Regression coefficients of true breeding values on genomic estimated breeding values for the validation animals (The subscripts are the standard deviations of 10 replicates)

**Trait**	**Data type**	**Single-trait**				**Multiple-trait**			
		**b**_**GEBV_m**_	**b**_**GEBV_mc**_	**b**_**GEBV_s**_	**b**_**GEBV_sc**_	**b**_**GEBV_m**_	**b**_**GEBV_mc**_	**b**_**GEBV_s**_	**b**_**GEBV_sc**_
I	No missing for both traits	1.105_0.016_	1.002_0.015_	1.108_0.017_	1.006_0.016_	1.105_0.016_	1.003_0.015_	1.112_0.017_	1.008_0.016_
	Missing for trait I	1.066_0.029_	0.959_0.032_	1.071_0.034_	0.964_0.037_	1.089_0.029_	0.980_0.031_	1.084_0.046_	0.975_0.048_
II	No missing for both traits	1.240_0.049_	0.875_0.038_	1.486_0.060_	1.048_0.054_	1.250_0.049_	0.880_0.038_	1.516_0.059_	1.069_0.055_
	Missing for trait II	1.212_0.116_	0.848_0.089_	1.444_0.209_	1.010_0.156_	1.190_0.067_	0.832_0.042_	1.567_0.121_	1.095_0.082_

b_GEBV_: the regression coefficient of true breeding values (TBV) on the estimated breeding values (EBVs) for the validation animals.

GEBV_m: the genomic estimated breeding value (GEBV) predicted using the EBV response variable calculated from the conventional multi-trait linear mixed model.

GEBV_s: the GEBV predicted using the EBV response variable calculated from the conventional single-trait linear mixed model.

GEBV_mc: GEBV_m divided by the average reliability of EBV for the reference animals.

GEBV_sc: GEBV_s divided by the average reliability of EBV for the reference animals.

## Discussion

The main advantage of MTGM over STGM is that MTGM uses information from genetically correlated traits [10,11]. The present study showed that MTGM gave more accurate GEBVs than STGM for the trait with low heritability and for the trait that had missing data when the data for the genetically correlated traits were complete. Hayashi and Iwata [12] reported that, compared to single-trait analysis, accuracy was increased with multi-trait analysis for a low heritability trait (h^2^ = 0.1) that had a high genetic correlation (0.7) with a high heritability trait (h^2^ = 0.8), using Bayesian variable selection models. In the present study, MTGM was favorable for the trait with a small number of records, which is very important in practical breeding programs because phenotype information for all traits of interest is often not available for all the animals in a reference population. For example, there is usually a limited amount of data for traits that are difficult or expensive to measure, such as carcass, feed efficiency, and disease traits. The accuracies of GEBVs obtained using a STGM will be low for traits with limited phenotypic data. By using information from the correlated and more easily measured traits, a MTGM will improve the accuracies of the GEBVs that are obtained. However, a MTGM will not be distinctly better than a STGM for traits with high heritability and for traits whose complete phenotypic data are available. This result was congruent with the findings of Hayashi and Iwata [12] who reported that multiple-trait and single-trait analyses made no difference to the prediction accuracy for a trait with high heritability (h^2^ = 0.8).

In this study, for the validation animals with no missing data, the reliability of GEBVs for the high heritability trait (h^2^ = 0.3) ranged from 0.873 to 0.874, and for low heritability trait (0.05) the reliability of GEBVs ranged from 0.718 to 0.756. However, the reliability of EBVs for the same animals ranged from 0.348 to 0.350 for the high heritability trait and from 0.182 to 0.229 for low heritability trait. These results showed that the reliability of GEBV was much higher than the reliability of EBV, and there were smaller differences in the reliability of GEBV between the low heritability and high heritability traits compared with the differences in the reliability of EBV. Su *et al.*[9] also reported relatively small difference in reliability of GEBV between milk (high heritability) and fertility (low heritability) in a Danish Holstein population. The small difference in reliability of GEBV for the validation animals between low and high heritability traits indicated that genetic evaluation using genomic prediction is relatively more beneficial for the trait with low heritability, particularly when no records are available for the candidate animals and their offspring (e.g., the pre-selection of young bulls for progeny testing). Therefore, compared with selection based on conventional EBV, genomic selection makes it relatively easier to improve functional traits such as udder health and fertility by reducing the cost of inputs [34], and to obtain a balanced genetic progress between functional traits and production traits.

In the present study, EBVs estimated from a single-trait or multiple-trait model (based on pedigree information) were used as response variables for genomic prediction. The results indicated that the reliability of GEBVs using EBV_m as the response variable were slightly higher than their reliability using EBV_s, except for trait II with no missing data. The reliability of GEBVs using EBV_m improved by 0.1% to 3.5% points compared with their reliability using EBV_s, because, in the multi-trait model, information about the correlated trait was used. Because the correlated trait data were used to estimate EBV_m, it can be argued that a multiple-trait model will be better than a single-trait model for genomic prediction when EBV_m is used as the response variable. The results from this study showed that even when the response variable was obtained from the multiple-trait model, the reliability of genomic prediction using MTGM was further improved over the reliability of STGM. A possible reason could be that the use of information from the correlated trait may not be exactly the same in the conventional BLUP and GBLUP methods. Thus, additional information from the correlated trait may be available for genomic prediction of the target trait, even though the response variable was obtained using the information of the correlated trait. A more important reason is that the prediction of GEBVs for the validation animals can use the information of the correlated trait directly through the covariance structure among the traits.

In the simulations, all the phenotypic records were available for the estimation of EBVs. The data with missing records for genomic prediction were generated by masking some of the EBVs. In a real world application however, missing phenotypes cannot be used to estimate EBVs. Therefore, in the scenarios with missing records, the prediction accuracy that was obtained in this study could be higher than the accuracy expected in the real world. On the other hand, the gain from using a multi-trait model in a real scenario with missing records might be larger than the gain from the simulated scenario, because the amount of information of the related trait in relation to the trait of interest would be relatively larger.

In this study, a GBLUP model was used for multiple-trait genomic prediction. Compared with the Bayesian variable selection models, the advantages of the multiple-trait GBLUP model are its low computational demand and its straightforward implementation using existing methodologies and standard linear mixed model software. However, the GBLUP model assumes that the effects of all the SNPs have the same normal distribution for a trait, and that the covariance between traits is the same for all SNP effects. This assumption may be not very appropriate and could limit the advantage of the GBLUP multiple-trait genomic prediction method. Hayashi and Iwata [12] used Bayesian variable selection models for multiple-trait genomic prediction. The Bayesian models assume a proportion of the SNP markers have an effect while others have a null effect, which describes the property of SNP effects more appropriately. However, their models assume a SNP either has an effect on all traits in the model or has no effect on any of the traits, and that the covariance between traits is the same for all SNP effects. In a real life scenario, different traits are affected by different sets of genes, and some genes have effects on more than one trait. More sophisticated models that can account for these features are required to fully exploit the advantages of multiple-trait genomic prediction.

## Conclusions

The results reported here suggest that a MTGM can improve the accuracy of genomic predictions, especially for low heritability traits and for traits with only a small amount of phenotype data. The EBV response variables derived from the multiple-trait and single-trait models had a relatively small influence on the reliability of GEBVs for the trait without missing data. However, for the trait with missing data, the response variable obtained from the multiple-trait model gave better genomic predictions than the response variable obtained from the single-trait model. Even when response variables derived from the multiple-trait model were used, the genomic prediction using MTGM still generated GEBVs with higher reliability than the GEBVs generated using STGM.

## Competing interests

The authors declare that they have no competing interests.

## Authors’ contributions

GG and FZ performed the data analyses and drafted the manuscript. YW assisted in the study design. GS and YZ reviewed the manuscript. LD and GS conceived and designed the study as well as co-supervised the work. All authors read and approved the final manuscript.
